# Poly[[μ_3_-*N*,*N*′-bis­(3-pyridylmeth­yl)­thio­urea-κ^3^ *N*:*N*′:*S*]iodidocopper(I)]

**DOI:** 10.1107/S1600536808031188

**Published:** 2008-10-15

**Authors:** Shi-Shen Zhang

**Affiliations:** a Department of Applied Chemistry, Zhejiang Sci-Tech University, Hang Zhou, 310018, People’s Republic of China

## Abstract

In the title coordination polymer, [CuI(C_13_H_14_N_4_S)]_*n*_, the Cu^I^ atom is coordinated by two N atoms from two *N*,*N*′-bis­(3-pyridylmeth­yl)thio­urea ligands, as well as by the S atom of a third ligand and an I atom to confer a distorted tetra­hedral coordination at the metal centre. The coordination bonds give rise to a layer structure parallel to (010).

## Related literature

For related literature, see: Li *et al.* (2002 ▶); Zhang *et al.* (2006 ▶).
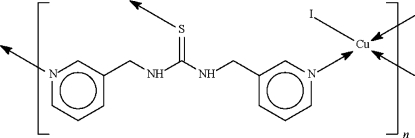


## Experimental

### 

#### Crystal data

[CuI(C_13_H_14_N_4_S)]
*M* *_r_* = 448.78
Monoclinic, 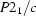

*a* = 13.3610 (10) Å
*b* = 8.3673 (7) Å
*c* = 14.2686 (11) Å
β = 102.001 (2)°
*V* = 1560.3 (2) Å^3^
*Z* = 4
Mo *K*α radiation
μ = 3.51 mm^−1^
*T* = 294 (2) K
0.20 × 0.15 × 0.12 mm

#### Data collection

Bruker SMART CCD diffractometer
Absorption correction: multi-scan (*SADABS*; Sheldrick, 1996 ▶) *T*_min_ = 0.541, *T*_max_ = 0.678
8191 measured reflections
2750 independent reflections
2418 reflections with *I* > 2σ(*I*)
*R*_int_ = 0.020

#### Refinement

*R*[*F* ^2^ > 2σ(*F* ^2^)] = 0.026
*wR*(*F* ^2^) = 0.062
*S* = 1.06
2750 reflections
181 parameters
H-atom parameters constrained
Δρ_max_ = 0.73 e Å^−3^
Δρ_min_ = −0.28 e Å^−3^

### 

Data collection: *SMART* (Bruker, 1998 ▶); cell refinement: *SAINT* (Bruker, 1998 ▶); data reduction: *SAINT*; program(s) used to solve structure: *SHELXS97* (Sheldrick, 2008 ▶); program(s) used to refine structure: *SHELXL97* (Sheldrick, 2008 ▶); molecular graphics: *SHELXTL* (Sheldrick, 2008 ▶); software used to prepare material for publication: *SHELXTL*.

## Supplementary Material

Crystal structure: contains datablocks I, global. DOI: 10.1107/S1600536808031188/ng2496sup1.cif

Structure factors: contains datablocks I. DOI: 10.1107/S1600536808031188/ng2496Isup2.hkl

Additional supplementary materials:  crystallographic information; 3D view; checkCIF report

## Figures and Tables

**Fig. 1.**
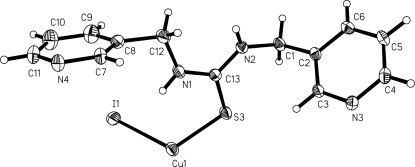
The asymmetric unit of the title compound showing 30° probability ellipsoids.

**Fig. 2.**
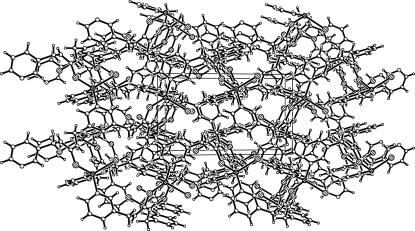
The crystal packing of the title compound.

## supplementary crystallographic information

### Comment

Flexible ligand has been considered as one of the most important type of organic ligand for their flexibility and conformational freedom allow for greater structural diversity. *N*,*N*'-bis(3-pyridylmethyl)thiurea, as one kind of those ligand, has usually been used to construct a great variety of structurally interesting entities. such as helix, macrocycle (Zhang *et al.*, 2006; Li *et al.*, 2002).

The asymmetric unit of the title compound (I) is illustrated in Fig. 1. Single-crystal X-ray diffraction shows that the asymmetric unit contains one Cu crystallographically nonequivalent atom. The Cu(I) atom coordinated by two N atoms from two N,N'-bis(3-pyridylmethyl)thiourea ligands as well as by the S atom of a third ligand to confer a tetrahedral geometry at the metal center. The Cu atom coordination by two N atoms to form a one-dimensional helix, and is then linked by the bond of Cu atom and S atom to extend to a two-dimensional structure. The crystal packing is stabilized by intermolecular π–π stacking interaction (Fig. 2).

### Experimental

a mixture of CuI (0.038 g, 0.2 nmol) and *N*,*N*'-bis(3-pyridylmethyl)thiurea (0.026 g, 0.1 nmol) in mole ratio of 2:1 in acetonitrile (6 cm^3^) was sealed in 15 cm^3^ Teflon-lined reactor and heated to 110°C for 10 h and then cooled to room temperature at a rate of 5°C/h. the yellow block crystal was obtianed in the yield of 35%.

The web of checkcif show one Alert level B (Hirshfeld Test Diff (M—*X*) I1 – Cu1.. 43.03 su), we think this is the result of the sightly distorted I atom for his unidentate coordination model.

### Refinement

H atoms were positioned geometrically and refined using a riding model, with C—H = 0.93 Å(aromatic) or 0.97 Å(aliphatic) and N—H = 0.86 Å, and with *U*_iso_(H) = 1.2*U_eq_*(C,*N*)

### Crystal data

**Table:**

[CuI(C_13_H_14_N_4_S)]	*F*(000) = 872
*M**_r_* = 448.78	*D*_x_ = 1.910 Mg m^−^^3^
Monoclinic, *P*2_1_/*c*	Mo *K*α radiation, λ = 0.71073 Å
Hall symbol: -P 2ybc	Cell parameters from 3234 reflections
*a* = 13.361 (1) Å	θ = 2.8–27.7°
*b* = 8.3673 (7) Å	µ = 3.51 mm^−^^1^
*c* = 14.2686 (11) Å	*T* = 294 K
β = 102.001 (2)°	Block, yellow
*V* = 1560.3 (2) Å^3^	0.20 × 0.15 × 0.12 mm
*Z* = 4	

### Data collection

**Table:**

Bruker SMART CCD diffractometer	2750 independent reflections
Radiation source: fine-focus sealed tube	2418 reflections with *I* > 2σ(*I*)
graphite	*R*_int_ = 0.020
φ and ω scans	θ_max_ = 25.0°, θ_min_ = 2.8°
Absorption correction: multi-scan (SADABS; Sheldrick, 1996)	*h* = −12→15
*T*_min_ = 0.541, *T*_max_ = 0.678	*k* = −9→9
8191 measured reflections	*l* = −15→16

### Refinement

**Table:**

Refinement on *F*^2^	Primary atom site location: structure-invariant direct methods
Least-squares matrix: full	Secondary atom site location: difference Fourier map
*R*[*F*^2^ > 2σ(*F*^2^)] = 0.026	Hydrogen site location: inferred from neighbouring sites
*wR*(*F*^2^) = 0.062	H-atom parameters constrained
*S* = 1.06	*w* = 1/[σ^2^(*F*_o_^2^) + (0.0275*P*)^2^ + 0.8144*P*] where *P* = (*F*_o_^2^ + 2*F*_c_^2^)/3
2750 reflections	(Δ/σ)_max_ = 0.001
181 parameters	Δρ_max_ = 0.73 e Å^−^^3^
0 restraints	Δρ_min_ = −0.28 e Å^−^^3^

### Special details

**Table:**

Geometry. All e.s.d.'s (except the e.s.d. in the dihedral angle between two l.s. planes) are estimated using the full covariance matrix. The cell e.s.d.'s are taken into account individually in the estimation of e.s.d.'s in distances, angles and torsion angles; correlations between e.s.d.'s in cell parameters are only used when they are defined by crystal symmetry. An approximate (isotropic) treatment of cell e.s.d.'s is used for estimating e.s.d.'s involving l.s. planes.
Refinement. Refinement of *F*^2^ against ALL reflections. The weighted *R*-factor *wR* and goodness of fit *S* are based on *F*^2^, conventional *R*-factors *R* are based on *F*, with *F* set to zero for negative *F*^2^. The threshold expression of *F*^2^ > σ(*F*^2^) is used only for calculating *R*-factors(gt) *etc*. and is not relevant to the choice of reflections for refinement. *R*-factors based on *F*^2^ are statistically about twice as large as those based on *F*, and *R*- factors based on ALL data will be even larger.

### Fractional atomic coordinates and isotropic or equivalent isotropic displacement parameters (Å^2^)

**Table:**

	*x*	*y*	*z*	*U*_iso_*/*U*_eq_	
C1	0.4137 (2)	0.8305 (4)	0.3317 (2)	0.0340 (7)	
H1A	0.4469	0.7269	0.3368	0.041*	
H1B	0.4581	0.9036	0.3739	0.041*	
C2	0.4006 (2)	0.8893 (3)	0.2301 (2)	0.0290 (6)	
C3	0.3491 (2)	1.0308 (3)	0.2012 (2)	0.0315 (7)	
H3	0.3220	1.0879	0.2460	0.038*	
C4	0.3768 (2)	1.0054 (4)	0.0489 (2)	0.0361 (7)	
H4	0.3685	1.0441	−0.0134	0.043*	
C5	0.4292 (2)	0.8663 (4)	0.0718 (2)	0.0402 (7)	
H5	0.4565	0.8122	0.0260	0.048*	
C6	0.4415 (2)	0.8065 (4)	0.1630 (2)	0.0376 (7)	
H6	0.4770	0.7115	0.1795	0.045*	
C7	−0.0360 (2)	0.7596 (4)	0.4500 (2)	0.0379 (7)	
H7	−0.0490	0.7921	0.3863	0.045*	
C8	0.0621 (2)	0.7151 (4)	0.4916 (2)	0.0372 (7)	
C9	0.0813 (3)	0.6670 (5)	0.5858 (3)	0.0521 (9)	
H9	0.1469	0.6365	0.6166	0.063*	
C10	0.0020 (3)	0.6648 (5)	0.6337 (3)	0.0571 (10)	
H10	0.0130	0.6307	0.6970	0.068*	
C11	−0.0934 (3)	0.7134 (4)	0.5874 (2)	0.0445 (8)	
H11	−0.1460	0.7147	0.6210	0.053*	
C12	0.1456 (2)	0.7225 (4)	0.4347 (3)	0.0413 (8)	
H12A	0.1909	0.6314	0.4502	0.050*	
H12B	0.1157	0.7194	0.3667	0.050*	
C13	0.2875 (2)	0.9112 (3)	0.4274 (2)	0.0309 (6)	
Cu1	0.26574 (3)	1.19634 (5)	0.57370 (3)	0.04022 (12)	
N1	0.20322 (18)	0.8710 (3)	0.45837 (19)	0.0397 (6)	
H1	0.1810	0.9377	0.4952	0.048*	
N2	0.31731 (18)	0.8164 (3)	0.36364 (17)	0.0317 (6)	
H2	0.2765	0.7407	0.3393	0.038*	
N3	0.33636 (18)	1.0893 (3)	0.11266 (18)	0.0328 (6)	
N4	−0.11373 (19)	0.7591 (3)	0.4956 (2)	0.0396 (6)	
S3	0.35264 (6)	1.07993 (10)	0.46956 (6)	0.0397 (2)	
I1	0.24731 (2)	1.03780 (3)	0.728706 (18)	0.05208 (10)	

### Atomic displacement parameters (Å^2^)

**Table:**

	*U*^11^	*U*^22^	*U*^33^	*U*^12^	*U*^13^	*U*^23^
C1	0.0309 (16)	0.0383 (16)	0.0344 (17)	0.0077 (12)	0.0101 (13)	−0.0001 (13)
C2	0.0242 (14)	0.0350 (16)	0.0285 (16)	−0.0015 (12)	0.0072 (12)	−0.0040 (13)
C3	0.0323 (16)	0.0333 (16)	0.0322 (17)	−0.0006 (12)	0.0140 (13)	−0.0037 (13)
C4	0.0379 (17)	0.0451 (17)	0.0268 (16)	−0.0068 (14)	0.0102 (14)	−0.0002 (14)
C5	0.0462 (19)	0.0439 (18)	0.0348 (18)	0.0034 (15)	0.0184 (15)	−0.0077 (15)
C6	0.0418 (18)	0.0334 (16)	0.0396 (19)	0.0072 (13)	0.0134 (14)	−0.0025 (14)
C7	0.0335 (17)	0.0461 (18)	0.0370 (18)	−0.0018 (14)	0.0139 (14)	−0.0007 (15)
C8	0.0338 (17)	0.0328 (16)	0.047 (2)	−0.0057 (13)	0.0128 (15)	−0.0044 (15)
C9	0.0371 (19)	0.065 (2)	0.052 (2)	0.0022 (16)	0.0035 (16)	0.0058 (19)
C10	0.055 (2)	0.076 (3)	0.040 (2)	−0.0008 (19)	0.0097 (17)	0.0101 (19)
C11	0.0411 (19)	0.056 (2)	0.040 (2)	−0.0058 (16)	0.0171 (15)	0.0028 (17)
C12	0.0329 (17)	0.0378 (17)	0.056 (2)	−0.0043 (14)	0.0151 (15)	−0.0110 (16)
C13	0.0311 (16)	0.0309 (15)	0.0312 (16)	0.0012 (12)	0.0079 (13)	−0.0013 (13)
Cu1	0.0403 (2)	0.0416 (2)	0.0416 (2)	−0.00434 (17)	0.01496 (18)	−0.00960 (18)
N1	0.0378 (14)	0.0374 (14)	0.0495 (17)	−0.0068 (12)	0.0223 (13)	−0.0140 (13)
N2	0.0346 (14)	0.0321 (13)	0.0297 (13)	−0.0022 (10)	0.0097 (11)	−0.0049 (11)
N3	0.0324 (13)	0.0361 (13)	0.0311 (14)	−0.0004 (11)	0.0099 (11)	0.0023 (11)
N4	0.0324 (14)	0.0475 (16)	0.0412 (16)	−0.0010 (12)	0.0129 (12)	0.0011 (13)
S3	0.0369 (4)	0.0383 (4)	0.0488 (5)	−0.0085 (3)	0.0203 (4)	−0.0128 (4)
I1	0.07354 (19)	0.04215 (15)	0.04564 (16)	0.00823 (11)	0.02411 (12)	0.01044 (10)

### Geometric parameters (Å, °)

**Table:**

C1—N2	1.457 (4)	C9—H9	0.9300
C1—C2	1.506 (4)	C10—C11	1.370 (5)
C1—H1A	0.9700	C10—H10	0.9300
C1—H1B	0.9700	C11—N4	1.337 (4)
C2—C6	1.384 (4)	C11—H11	0.9300
C2—C3	1.388 (4)	C12—N1	1.464 (4)
C3—N3	1.332 (4)	C12—H12A	0.9700
C3—H3	0.9300	C12—H12B	0.9700
C4—N3	1.348 (4)	C13—N2	1.330 (4)
C4—C5	1.363 (4)	C13—N1	1.335 (4)
C4—H4	0.9300	C13—S3	1.701 (3)
C5—C6	1.371 (5)	Cu1—N3^i^	2.049 (2)
C5—H5	0.9300	Cu1—N4^ii^	2.099 (3)
C6—H6	0.9300	Cu1—S3	2.2852 (8)
C7—N4	1.335 (4)	Cu1—I1	2.6341 (5)
C7—C8	1.372 (4)	N1—H1	0.8600
C7—H7	0.9300	N2—H2	0.8600
C8—C9	1.374 (5)	N3—Cu1^iii^	2.049 (2)
C8—C12	1.512 (4)	N4—Cu1^ii^	2.099 (3)
C9—C10	1.376 (5)		
			
N2—C1—C2	113.3 (2)	C9—C10—H10	120.3
N2—C1—H1A	108.9	N4—C11—C10	122.4 (3)
C2—C1—H1A	108.9	N4—C11—H11	118.8
N2—C1—H1B	108.9	C10—C11—H11	118.8
C2—C1—H1B	108.9	N1—C12—C8	108.8 (2)
H1A—C1—H1B	107.7	N1—C12—H12A	109.9
C6—C2—C3	117.6 (3)	C8—C12—H12A	109.9
C6—C2—C1	121.3 (3)	N1—C12—H12B	109.9
C3—C2—C1	121.1 (2)	C8—C12—H12B	109.9
N3—C3—C2	123.6 (3)	H12A—C12—H12B	108.3
N3—C3—H3	118.2	N2—C13—N1	118.0 (3)
C2—C3—H3	118.2	N2—C13—S3	122.2 (2)
N3—C4—C5	122.7 (3)	N1—C13—S3	119.8 (2)
N3—C4—H4	118.6	N3^i^—Cu1—N4^ii^	108.54 (10)
C5—C4—H4	118.6	N3^i^—Cu1—S3	106.38 (7)
C4—C5—C6	119.6 (3)	N4^ii^—Cu1—S3	110.01 (8)
C4—C5—H5	120.2	N3^i^—Cu1—I1	109.34 (7)
C6—C5—H5	120.2	N4^ii^—Cu1—I1	103.57 (7)
C5—C6—C2	119.1 (3)	S3—Cu1—I1	118.69 (3)
C5—C6—H6	120.4	C13—N1—C12	125.3 (2)
C2—C6—H6	120.4	C13—N1—H1	117.4
N4—C7—C8	123.9 (3)	C12—N1—H1	117.4
N4—C7—H7	118.0	C13—N2—C1	125.2 (3)
C8—C7—H7	118.0	C13—N2—H2	117.4
C7—C8—C9	118.0 (3)	C1—N2—H2	117.4
C7—C8—C12	120.1 (3)	C3—N3—C4	117.3 (3)
C9—C8—C12	121.9 (3)	C3—N3—Cu1^iii^	122.7 (2)
C8—C9—C10	118.8 (3)	C4—N3—Cu1^iii^	119.9 (2)
C8—C9—H9	120.6	C7—N4—C11	117.2 (3)
C10—C9—H9	120.6	C7—N4—Cu1^ii^	123.0 (2)
C11—C10—C9	119.5 (3)	C11—N4—Cu1^ii^	119.4 (2)
C11—C10—H10	120.3	C13—S3—Cu1	106.95 (10)

Symmetry codes: (i) *x*, −*y*+5/2, *z*+1/2; (ii) −*x*, −*y*+2, −*z*+1; (iii) *x*, −*y*+5/2, *z*−1/2.
